# Stoichiometric Thiol Redox Proteomics for Quantifying Cellular Responses to Perturbations

**DOI:** 10.3390/antiox10030499

**Published:** 2021-03-23

**Authors:** Nicholas J. Day, Matthew J. Gaffrey, Wei-Jun Qian

**Affiliations:** Pacific Northwest National Laboratory, Biological Sciences Division, Richland, WA 99352, USA; nicholas.day@pnnl.gov (N.J.D.); matthew.gaffrey@pnnl.gov (M.J.G.)

**Keywords:** redox proteomics, cysteine, redox post-translational modifications, thiol proteome

## Abstract

Post-translational modifications regulate the structure and function of proteins that can result in changes to the activity of different pathways. These include modifications altering the redox state of thiol groups on protein cysteine residues, which are sensitive to oxidative environments. While mass spectrometry has advanced the identification of protein thiol modifications and expanded our knowledge of redox-sensitive pathways, the quantitative aspect of this technique is critical for the field of redox proteomics. In this review, we describe how mass spectrometry-based redox proteomics has enabled researchers to accurately quantify the stoichiometry of reversible oxidative modifications on specific cysteine residues of proteins. We will describe advancements in the methodology that allow for the absolute quantitation of thiol modifications, as well as recent reports that have implemented this approach. We will also highlight the significance and application of such measurements and why they are informative for the field of redox biology.

## 1. Introduction

### 1.1. Post-Translational Modifications (PTMs) of Protein Cysteine Thiols 

**Figure 1:**
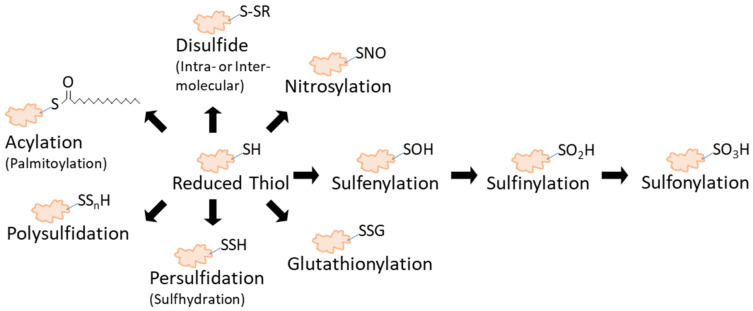
Cysteine thiol modifications. Reduced thiols can be subjected to multiple types of modifications. In this cartoon, disulfide formation includes those that form intramolecularly, or within the same protein, or intermolecularly through exchange with a sulfur group on another protein or molecule. Sulfinylation and sulfonylation occur as subsequent, irreversible oxidative modifications of cysteine thiols that are sulfenylated. Note that persulfidation is also referred to as sulfhydration. Palmitoylation is shown here as a representative modification of acylation, which is the covalent linking of an acyl chain to a cysteine; however, acylation can include other molecules besides 16C palmitate. With the exception of sulfinylation and sulfonylation, all modifications illustrated here are considered reversible.

The activation or integration of proteins in signaling pathways results from regulatory mechanisms that control their function. These include PTMs of amino acids by way of the inclusion of molecules or chemical groups that add to the diversity of a protein’s function in signaling pathways [1]. Cysteine is one of the least abundant amino acids in proteins [2,3], yet it plays an important role in modulating protein structure and activity. The thiol group in the sidechain of cysteine possesses chemical and physical properties that contribute to the important role of this amino acid in many biochemical reactions [4,5,6]. As a result, cysteines are often found in protein domains associated with catalysis, metal coordination, and structural changes and also participate in redox sensing [7]. The deprotonation of the thiol group under physiological conditions leads to the formation of a reactive thiolate group that is susceptible to further modifications. In particular, reactive oxygen, nitrogen, or sulfur species are among the types of molecules that can react with thiolates to modify proteins, often via an oxidation-reduction (redox) reaction [8]. The types of reversible thiol modifications include disulfide formation [9], nitrosylation [10], sufenylation [11], glutathionylation [12], persulfidation [13], polysulfidation [13], and palmitoylation [14] (Figure 1). It is also worth noting that some of these oxidative modifications may function as intermediates to prime the cysteine for another modification, such as nitrosylation or sulfenylation, which can later be switched to glutathionylation [15]. In addition, sulfenylated thiols are capable of further irreversible oxidation to form stable sulfinic or sulfonic acids (Figure 1) that are considered markers of oxidative stress and damage [16,17,18]. The field has held a longstanding view that sulfinic acids are irreversible; however, recent work has shown that sulfiredoxins function as sulfinic acid reductases for numerous proteins beyond peroxiredoxins [19]. Interestingly, metabolites and other cysteine-reactive natural products can also function as strong electrophilic compounds and form adducts with thiols that impact target protein function [20,21,22,23,24]. This suggests that the current account of thiol-based modifications may be even broader than what is currently known and could continue to increase with future studies. With multiple types of thiol modifications, it should be noted that the so-called “oxidized thiols” exist as a combination of all reversible oxidative modifications. This review will emphasize the concept of “total oxidation”, which collectively represents all types of reversible thiol modifications. 

**Figure 2:**
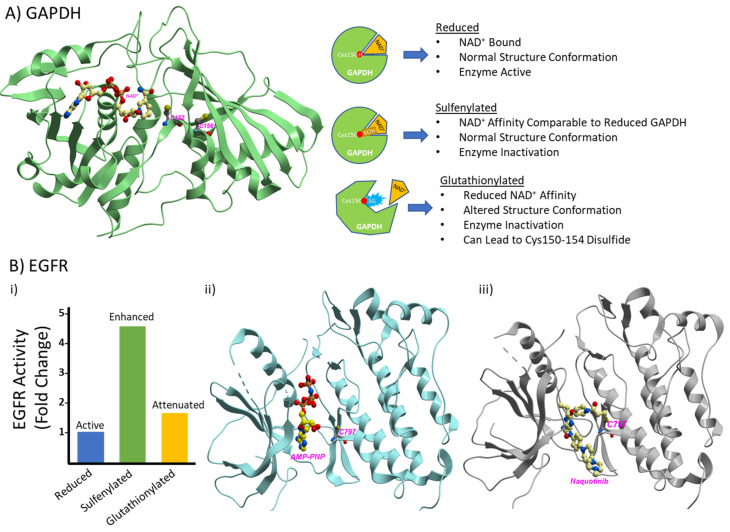
Thiol redox modifications at the molecular level. Two examples where thiol redox modifications alter the structural and functional properties of proteins are presented. (**A**) Human GAPDH monomer in complex with the nicotinamide adenine dinucleotide (NAD^+^) cofactor (PDB: 1u8f) [30]. Cysteines 152 and 156 are shown and labeled in magenta and highlight the close proximity of Cys152 to the NAD^+^ binding pocket. Note that the catalytic cysteine in GAPDH is Cys152 in humans and Cys150 in mice, rats, or rabbits. Simplified cartoons of findings from rabbit GAPDH Cys150 [27] when reduced, sulfenylated, and glutathionylated are shown to the right of the structure. Glutathionylation of GAPDH active site cysteine can also lead to the formation of an intramolecular disulfide that causes GAPDH to inactivate and aggregate but protects against irreversible oxidation. (**B**) (**i**) Representative bar plot of EGFR (epidermal growth factor receptor) activity when the Cys797 in the active site is reduced, sulfenylated, or glutathionylated. The plot represents trends as fold change based on the data observed in [31]. (**ii**) Wild-type human EGFR kinase domain (PDB: 3vjo) [32] with Cys797 shown and labeled in magenta. Adenosine 5′-(β,γ-imino)triphosphate (AMP-PNP), a non-hydrolysable analog of ATP, is also shown in the molecule to highlight Cys797’s close proximity to the catalytic site of EGFR. (**iii**) T790M mutant human EGFR in complex with the inhibitor naquotinib (PDB: 5y9t) [33], which is covalently linked to Cys797 and is used to treat cancer. The efficacy of inhibitors that covalently bind to Cys797 can be impacted by the oxidation of Cys797. Molecular images were generated with ICM-Browser (http://www.molsoft.com/icm_browser.html (accessed on 9 December 2020)).

While it is known that there are multiple types of thiol redox modifications, understanding their physiological significance is also critical to determining their importance in the cellular environment. As an illustration, glyceraldehyde-3-phosphate dehydrogenase (GAPDH), an important enzyme in the glycolytic pathway, is susceptible to multiple types of thiol redox modifications, such as nitrosylation at the active site residue Cys150 of mouse GAPDH [25]. A more recent study has found that the corresponding cysteine in human GAPDH, Cys152, is subject to sulfonic acid formation that irreversibly inactivates GAPDH [26]. Sulfenylation at the same residue in rabbit GAPDH in response to hydrogen peroxide exposure has also been reported [27], where the sulfenic acid promotes a reaction with reduced glutathione. The same study found that the glutathionylation of GAPDH results in the dissociation of the NAD^+^ cofactor, causing enzyme inactivation, changes in tertiary structure, and a reduction in the thermal stability of the protein (Figure 2A). Interestingly, persistent glutathionylation has been shown to be removed through the formation of an intramolecular disulfide between the active site and neighboring cysteines, resulting in GAPDH aggregation [28]. The physiological relevance of these aggregates continues to be investigated; however, some studies point to different effects, such as mitochondrial dysfunction [29]. These findings strongly suggest that thiol redox modifications act as critical regulators of GAPDH’s structural and functional properties (Figure 2A). 

Another example is the epidermal growth factor receptor (EGFR), which is an important receptor tyrosine kinase for signaling in development and metabolism and has become an important target for treating cancers. Exposure of EGFR to moderate levels of hydrogen peroxide (0.05–10 µM) leads to sulfenylation at Cys797 in the catalytic ATP-binding site, resulting in enhanced tyrosine kinase activity [31,34] (Figure 2Bi). Molecular modeling later found that this enhancement of EGFR activity is likely due to the introduction of a new hydrogen bond and electrostatic interactions with a neighboring catalytic loop in the active site [35]. Glutathionylation observed at Cys797 reduces the sulfenylation-based enhancement of EGFR kinase activity [31] (Figure 2Bi) and plays a role in the internalization and translocation of EGFR to the nucleus [36]. Cys797 is in close proximity to the catalytic site of the EGFR kinase domain where ATP binds (Figure 2Bii, ATP analog bound EGFR) and has been the subject of intensive research as numerous tyrosine kinase inhibitors target this site as a treatment for cancer (Figure 2Biii, inhibitor bound EGFR). A previous study has found that oxidative modifications interfere with the efficacy of drugs that target or react with Cys797 in this site [37]. Thiol redox modifications can change the steric space of the active site, making it difficult for substrates and inhibitors to dock, especially with a bulky group like glutathione [37,38]. These findings demonstrate that thiol redox modifications play a major role in regulating the active site dynamics of EGFR, and likely many other proteins. These examples highlight the diversity of redox modifications as an important focal point of research in the field of redox biology.

The sensitivity of thiol groups towards changes in the surrounding environment has led them to be characterized as sensors or sentinels of the redox environment in the cell [6,39,40]. This is due in part to oxidized or reduced thiols that serve as “on” or “off” switches to activate pathways sensitive to changes in the redox state under physiological or oxidative stress conditions [3,6,39,41,42]. This can lead to the modulation of protein activity and differential functions, resulting in alternate signaling outcomes [43]. As a result, the oxidation levels of critical residues that impact signaling outcomes may prove to be useful markers of a disease in cells. From a structural perspective, as noted by Go and Jones, thiol redox modifications can be classified into four different types of switches that have a modulatory effect: on-off (protein in-/activation), interaction (binding), allosteric (regulates activity), and thiolation (alternate function) [44]. As some thiol-based redox modifications are reversible, this can permit rapid activation or inactivation of signaling pathways [45], leading to their transient activity [46,47]. One example is the transient receptor potential (TRP) family of Ca^2+^ cation channels that are found in photoreceptors and other cell types. Oxidation of redox-sensitive cysteines in TRP proteins can induce Ca^2+^ fluxes that can quickly up- or down-regulate the activity of other signaling pathways [48,49]. Many pathways that are impacted by redox signaling include cell death [50], cell differentiation [51], aging [52], inflammation [53], and metabolism [54]. To ensure that these, and other redox-sensitive pathways, are properly executed, there are a variety of factors that work to prevent the overoxidation of proteins and maintain a balanced redox environment.

### 1.2. Maintenance of the Cellular Redox State

Since thiol oxidation plays a critical role in the activity of a variety of biological processes, it is also important to consider how these oxidative modifications are regulated. While thiol groups are important transducers of redox-mediated signaling, they are also utilized in antioxidant systems [55,56]. Thiol-containing cellular antioxidant systems contain small molecules [57] and enzymes [58] that are used to maintain the homeostasis of the redox state. Together, these components comprise the antioxidant response systems that attempt to prevent oxidative stress of the cell [59]. One example of these small antioxidant molecules is glutathione, which is an abundant, thiol-containing compound that plays an important role in maintaining the redox balance in cells [60]. Glutathione helps to maintain a reduced environment through its formation of reversible covalent bonds with thiol groups on proteins or other molecules. Glutathionylated sites can be reversed to free thiols through enzymes known as glutaredoxins, where the N-terminal active site uses thiol groups to catalyze this process [43,61,62,63]. Other enzymes that maintain the redox environment through the unique properties of thiol groups include thioredoxins that can reverse the sulfenylation or nitrosylation of thiol groups on proteins [64,65,66,67]. Together, peroxiredoxins and sulfiredoxins form an axis that is also important for regulating the balance of peroxides in the cell [68,69,70]. In order to respond to changes in the redox state of the cell, it should be noted that the synthesis of some of these enzymes is driven by transcription factors that are sensitive to changes in the redox environment [59,71], namely the Nrf2 pathway. Taken together, the unique properties of thiol groups enable their use as both switches for signaling pathways as well as antioxidant systems that regulate the redox state of the cell.

**Figure 3:**
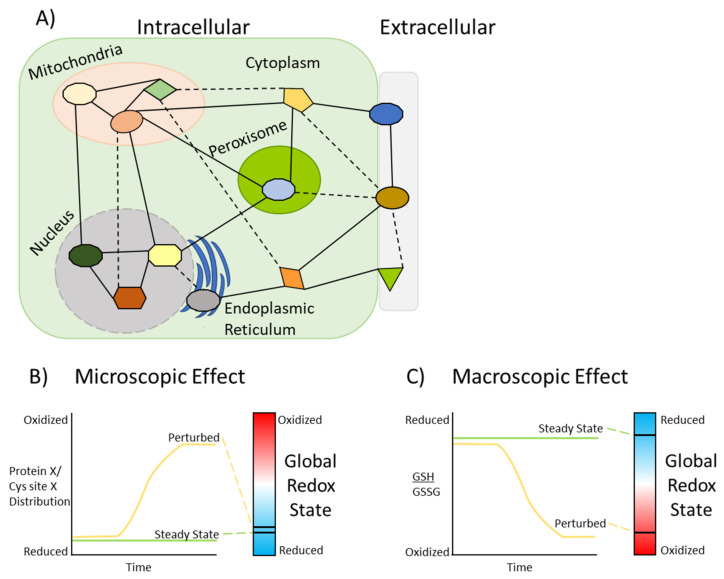
Modeling redox networks and redox state. (**A**) Simplified cartoon representing the redox circuits of a redox network present in the extracellular domain (e.g., receptors) and different intracellular compartments of a cell. The uniquely colored shapes represent different pathways, or circuits, that function in specific subcellular compartments. Interconnected pathways are denoted by solid lines that link the shapes, while dashed lines represent transient interactions among circuits. (**B**) Plot illustrating the microscopic effect of thiol redox signaling on the global redox state. For a given protein (protein X) or redox-sensitive cysteine site (Cys site X), the distribution of oxoforms can vary between steady state and perturbed conditions. This plot shows that the majority of oxoforms of a protein or cysteine site are reduced under steady state conditions; however, in the presence of a perturbing agent, the distribution transitions to a majority of oxidized oxoforms. As noted by the heatmap to the right, the transition of the protein or Cys site to become more oxidized following a perturbation has a negligible effect on the overall redox state of the cell. Under homeostatic conditions, the transition of reversibly oxidized proteins and Cys sites to become more or less oxidized within a specific circuit may also have little impact on the global redox state. (**C**) Plot illustrating the macroscopic effects of thiol redox signaling. In this scenario, the ratio of reduced versus oxidized glutathione (GSH/GSSG) is monitored as a readout for the global redox state. Under steady state conditions, the global redox state is under a more reduced environment (more GSH than GSSG); however, a severe perturbation causes the global redox state to become more oxidized (more GSSG than GSH). This scenario demonstrates an oxidative stress; however, the same effect could be observed from a perturbation that causes reductive stress. In the context of the model in (**A**), a macroscopic effect may be observed across multiple circuits, as opposed to a microscopic effect observed in one circuit.

The increasing number of identified redox sensitive Cys sites has led researchers to broadly conceptualize the organizational structure of the redox state in the cell. Thiol redox signaling has been described as forming a network containing sensory and responsory components that facilitate redox homeostasis [72,73]. This network is governed by several underlying principles that have been outlined by Jones and Sies, who proposed the concept of a ‘redox code’ [74]. The redox code is comprised of four parts that help to explain how the redox network functions: (1) NAD and NADP systems are at the center of metabolic organization and bioenergetics needed for redox reactions; (2) metabolism controls the kinetics of redox switches on proteins, which control protein structure, activity, interactions, and function; (3) redox sensing is spatiotemporally organized to control specific biological processes; and (4) redox networks form an adaptive system that governs the response to environmental stimuli [74]. Together, these principles of the redox code help to define the organized molecular logic of thiol redox signaling and how it enables a wide array of biological processes. At the cellular level, organization of the redox network is made up of individual circuits, or pathways, that integrate the principles of the redox code, which has been suggested for mitochondria [75]. Beyond proteins with thiol redox “switches”, cysteine/cystine- and glutathione-containing buffering and thioredoxin reductase systems are also important components of these circuits, as they control the reversible oxidation of thiols [76]. The redox circuit concept is extendable to other compartments of a cell, such as the cytoplasm, nucleus, and endoplasmic reticulum, which contain their own specific microenvironments and conditions for thiol redox signaling as indicated by their varying redox potentials [77,78,79] (Figure 3A). Compartmentalization organizes these circuits so that they are insulated from one another to ensure effective and coordinated signaling with minimal interference from other pathways [78]. Inter-compartment signaling is another potential function of the redox network (Figure 3A). Crosstalk between the redox signaling pathways in mitochondria and peroxisomes has been discussed, wherein these organelles function cooperatively to ensure the fitness of the cell and to regulate redox metabolism [80]. Genetic mutations, such as those that cause cancer, can cause metabolism and redox signaling circuits to become interconnected, and lead to undesirable effects that promote growth and resistance against therapies [81]. 

Since cells have multiple compartments with different redox characteristics and circuits, it can be difficult to evaluate the effects of a perturbation in an experimental context. Therefore, the distinction between the micro- and macroscopic effects of a perturbation on the thiol redox network needs to be considered in terms of the impact on overall redox state. Following a perturbation, thiol redox signaling occurs as a discrete signaling event, causing a localized effect without any change to the global redox state [75], which we define as a microscopic effect (Figure 3B). In this type of situation where only one circuit or pathway is affected, homeostasis may not be disrupted, especially if the perturbed circuit is well-insulated or does not crosstalk with other pathways. This model may also apply to basal state conditions, where thiol redox signaling occurs as necessary to activate specific circuits without disrupting the global redox state. On a macroscopic scale, perturbation to many redox circuits leads to a global change in redox state, which can be evaluated by monitoring the distribution of small molecule antioxidants, such as the ubiquitous molecule glutathione, through comparison of the ratio of reduced and oxidized forms (GSH/GSSG) [75] (Figure 3C). Understanding the distinction between these micro- or macroscopic effects and how they relate to global redox state following a perturbation can help to identify and characterize redox-sensitive pathways. 

Depending on what proteins are affected by a perturbation, downstream pathways may be activated directly or indirectly through relay mechanisms using intermediates [82], which adds to thiol redox network complexity (Figure 3A). From a systems biology perspective, it is important to consider the pleiotropic effects or the ‘duality’ of oxidants on cellular processes, as was highlighted with neuron development and cell fate in a recent review [83]. While thiol oxidation is important for underlying biological processes (microscopic effects), exposure to supraphysiological or excessive levels of oxidants leads to harmful or lethal effects (macroscopic effects). As severe perturbations are considered to induce stronger or alternate responses compared to mild stimuli, the profile or degree of protein oxidation may be dramatically different [84]. This is apparent when looking at the redox state or degree of protein oxidation, where severe perturbations induce stronger responses compared to mild or no stimuli. Using a photoinducible system to fine-tune reactive oxygen species (ROS) generation, a previous study found that varying levels of oxidative stress induced different response pathways and altered cell morphology [85]. More recently, a side-by-side comparison of exposure to different types of ROS-inducing nanoparticles led to different response pathways and varying levels of glutathionylation [86]. These studies suggest that thiol oxidation is dynamic, and that the thiol redox network can be perturbed in a variety of ways, resulting in varying types of responses. To better understand the physiological relevance of thiol oxidation and how it relates to thiol redox circuit activity, it is important to determine the levels of oxidative modifications at specific protein Cys residues. 

### 1.3. General Approaches for Measuring Redox PTMs

In the past, an array of different methods have been used to monitor protein oxidation [87]. Difference gel electrophoresis (DIGE) can indicate which proteins undergo changes in protein thiol oxidation under different conditions within the same gel [88]. By blocking free thiols and reducing reversibly oxidized thiols so that they can be labeled with fluorescently tagged alkylating agents (containing different fluorophores), users can observe the contrast in protein thiol oxidation from multiple samples. Alternatively, modified proteins can be probed by immunoblotting when using antibodies against a specific modification such as glutathionylation or nitrosylation [89]. Another early approach used ^35^S-labeled glutathione to identify proteins that undergo glutathionylation. This, coupled with 2D gel electrophoresis and mass spectrometry, enables users to identify new proteins that are glutathionylated [90]. The development of chemoselective probes that identify specific types of cysteine oxidative modifications and improvements in mass spectrometry (MS)-based proteomic methods has significantly advanced the field of redox proteomics [87,91]. Since thiol groups exhibit versatility in reactivity towards numerous types of redox-based modifications, this reactivity has been captured with different chemical probes for direct labeling [8,24,92,93,94,95]. For example, sulfenylation can be probed in vivo or in situ using dimedone [96], and nitrosylated proteins can be enriched using an organomercury resin [97]. Beyond direct probing, significant advances using indirect strategies for profiling the thiol redox proteome have been reported [98]. This involves blocking free thiol groups with an alkylating reagent such as N-Ethylmaleimide (NEM) or iodoacetamide (IAA), which is followed by the selective reduction of an oxidative modification, such as de-glutathionylation with glutaredoxin [86] or the reduction of nitrosylation by ascorbate [99]. To improve detection and reduce sample complexity and ionization suppression effects, oxidatively modified and labeled proteins can be enriched out of a cellular lysate as was demonstrated with the biotin switch technique (BST) by Jaffrey and colleagues in 2001 [100]. Using this technique, free thiols are initially blocked, and then reversibly oxidized thiols are selectively reduced and labeled with a biotin tag for enrichment by streptavidin-coated beads. Other strategies using the same methodology as BST have been developed with greater specificity and are described below (Section 2.3.2 and Section 2.3.3). These techniques enable thiol redox modifications to be profiled via MS to identify and quantify the small fractions of proteins that are modified among a greater population of unmodified proteins. In line with the scope of this review, we will discuss methods below that incorporate these labeling or enrichment strategies to quantitatively measure the stoichiometry of total oxidation of thiols in any given sample.

## 2. Stoichiometric Quantification of Thiol Redox Modifications Using Mass Spectrometry

### 2.1. PTM Stoichiometry

**Figure 4:**
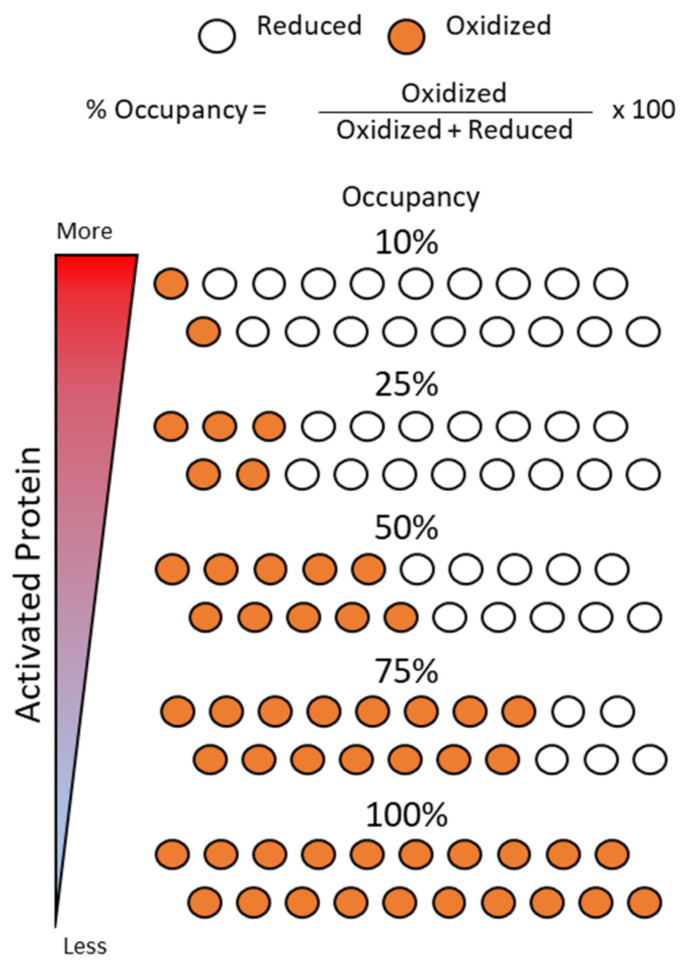
Thiol redox state and stoichiometry concept. For any given protein, the occupancy of oxidized versus reduced forms is determined by the ratio of oxidized over total abundance of protein analyzed (total sum of oxidized and reduced forms) and is used to interpret modification stoichiometry. Multiple scenarios of increasing thiol oxidation are illustrated to represent how the stoichiometry of oxidative modifications can change. Depending on the type of modification and protein, thiol redox modifications can activate or inactivate a protein’s function. As an example, the heatmap gauge on the left side of the figure illustrates how changes in thiol oxidation stoichiometry can correlate with the amount of activated protein, where more oxidation leads to less activated protein.

Quantitative PTM analyses offer greater insight into signaling pathways by providing precise measurements that distinguish changes between a perturbation and steady state. Mass spectrometry is a valuable tool for the field of redox proteomics as it enables both the identification and quantification of PTMs via downstream workflows. While it is well established that mass spectrometry can quantitatively measure protein abundance [101], it has also been adapted for the quantification of PTMs on a residue-by-residue basis. This allows for the determination of the fractional occupancy of a modification, which is expressed as the ratio of modified versus total residues observed [102,103] (Figure 4). The advantage of measuring site occupancy is that it offers a more precise representation of the contrast between modified and unmodified protein compared to fold change. Occupancy is used to conceptualize modification stoichiometry and aids in determining the significance of a modification at a specific site. For example, a fourfold increase in oxidation could be observed in low-occupancy sites (1% to 4%) as well as moderately occupied sites (25% to 100%) (Figure 4). As a result, the identification of key biologically relevant sites may be overlooked if left to analyses that focus on fold change measurements [102,103]. As discussed below, stoichiometric measurements provide a quantitative way of assessing the impact of a perturbation or to gain insight into the basal state PTMs in a model organism.

### 2.2. Applying Stoichiometry to Redox Proteomics

Depending on the biological effect of thiol oxidation for a given protein, significant changes in thiol oxidation stoichiometry could cause large changes in protein activity without the alteration of protein abundances (Figure 4). As a result, this metric helps to interpret the functional relevance of modifications [84,103]. In the thiol redox field, mass spectrometry enables the identification of redox-sensitive Cys sites and their modifications, as well as the quantification of modification stoichiometry to distinguish biologically significant sites. This ultimately leads researchers towards a better understanding of the network of redox-controlled protein thiol groups [104,105].

**Figure 5:**
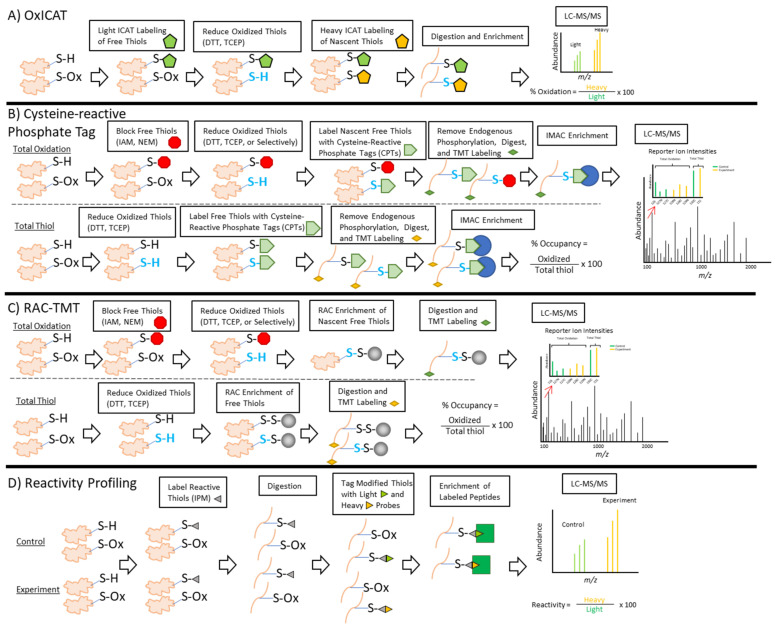
Simplified schematics of different quantitative redox proteomics approaches. Each schematic uses two proteins per scenario to represent the reduced (S-H) and oxidized (S-Ox) forms. Oxidized thiols represent any variety of reversible thiol modifications shown in Figure 1. Blue reduced thiol groups represent the formation of nascent free thiols following the reduction of oxidized thiols. In the cysteine-reactive phosphate tag and RAC (resin assisted capture) methods, total thiol samples are included as a separate reference channel for stoichiometric measurements (i.e., total thiol, representing all detectable thiols). Measurements are based on reporter ion intensities. (**A**) Labeling reduced and oxidized cysteines with isotope coded affinity tags (OxICAT). Free thiols are irreversibly labeled with isotopically light ICAT, followed by the reduction of oxidized thiols to free thiols that are labeled with isotopically heavy ICAT. Labeled proteins are then digested and enriched by streptavidin beads via the biotin group on the ICAT reagents. The enriched peptides are released by the cleavage of the biotin moiety and analyzed by mass spectrometry. The modification stoichiometry is determined by the ratio of a peptide’s corresponding heavy and light peptide intensities. (**B**) Free thiols are initially alkylated, and then oxidized thiols are totally or selectively reduced (if probing for a specific modification). Endogenous phosphorylation is removed to avoid the recovery of phosphorylated peptides during immobilized metal affinity chromatography (IMAC). Nascent free thiols are labeled with cysteine-reactive phosphate tags (CPTs), the proteins are digested, and the peptides are labeled with tandem mass tags (TMT). The CPT tags allow the peptides to be enriched by IMAC and are eluted off the column prior to analysis by mass spectrometry. The oxidation occupancy is determined by taking the ratio of reporter ion intensities in the oxidized and total thiol channels to evaluate the modification stoichiometry. It is ideal that each sample type (i.e., control, experiment, treated, untreated) should have its own total thiol channel for the appropriate measurement and comparison of redox state. (**C**) Similar to the approach in (**B**), free thiols are alkylated, and then oxidized thiols are reduced totally or selectively. Resin assisted capture (RAC) of nascent free thiols using a thiopropyl sepharose resin is followed by on-resin digestion and TMT labeling. Finally, peptides are eluted off the resin prior to their analysis by mass spectrometry. The oxidation occupancy is determined by taking the ratio of reporter ion intensities in the oxidized and total thiol channels to evaluate the modification stoichiometry. It is ideal that each sample type should have its own total thiol channel for the appropriate measurement and comparison of redox state. (**D**) Like OxICAT, thiol reactivity profiling uses isotopically light and heavy thiol-directed probes; however, these probes are designed to only label free thiols to identify and quantify reactive thiol groups in different types of samples (control or treated). Therefore, this method does not represent the oxidized cysteines, but rather cysteines that are reactive. The procedure begins by labeling free thiols with a probe called 2-iodo-N-(prop-2-yn-1-yl)acetamide) (IPM), which is followed by digestion. Thiol groups labeled by the IPM reagent are then tagged with isotopically light or heavy variants of an azido-UV-biotin tag and enriched by streptavidin-coated beads. The enriched peptides are eluted by UV due to the photocleavable linker in the azido-UV-biotin tag and analyzed by mass spectrometry. The reactivity is determined by the ratio of a peptide’s corresponding heavy and light peptide intensities.

While there are multiple approaches that make proteomics quantitative, the implementation of isobaric labeling has been effective for the multiplexed quantification of protein abundances in samples analyzed by mass spectrometry (Figure 5B,C). Two commonly used forms of isobaric labels include isobaric tags for relative and absolute quantification (iTRAQ) [106] and tandem mass tags (TMT) [107]. In this review, we will discuss TMT in further detail, but acknowledge that iTRAQ labeling is a valid alternative for stoichiometric measurements of protein modifications. During peptide fragmentation in MS/MS, the reporter region attached to the TMT tag is released to form a reporter ion. The intensity of a peptide’s corresponding reporter ion is used as a measurement to determine the occupancy of a modification, or the stoichiometry. This is expressed as a ratio of intensity values observed for a peptide in control and experimental samples. This method has been used to study different modifications, including phosphorylation [108], as well as thiol glutathionylation and oxidation [109]. The multiplexed capability of TMT labels is advantageous for profiling multiple types of oxidative thiol modifications within a sample. A recent report from our lab demonstrated that the stoichiometry of glutathionylation as well as total thiol oxidation in the sample could be measured together simultaneously [109]. This also permitted measurement of how much thiol oxidation was attributed to glutathionylation, which provides a more precise representation of the extent of this modification among all other possible forms of thiol oxidation. For simplicity, this review will focus on the discussion of the methods in Section 2.3 that quantitatively measure total oxidation of thiols (regardless of the reversible modification) in comparison to total thiol, which represents all thiols that are detectable in a sample (totally reduced by DTT, TCEP, etc.). We acknowledge that total oxidation measurements exclude irreversible modifications like sulfinylation and sulfonylation, as they are unable to be selectively reduced. However, a previous report has found that sulfinic and sulfonic acids make up a very small fraction that is negligible [110] during the analysis of the total oxidation of thiols.

### 2.3. Methods That Measure Stoichiometry in Thiol Redox Proteomics 

#### 2.3.1. OxICAT

The oxidative isotope-coded affinity tags approach, also known as oxICAT, was initially introduced in 2008, when Leichert and colleagues investigated the oxidation of thiol-containing proteins in *E. coli* in response to hypochlorite or hydrogen peroxide stress [111]. Briefly, the ICAT reagent is a chemical probe that labels reduced thiol groups due to its iodoacetamide moiety, which rapidly and irreversibly reacts with free thiols (Figure 5A). The power of this approach to resolve reduced versus oxidatively modified thiols lies in the difference between the light and isotopically heavy carbon linkers of the ICAT reagent. Once free thiols are labeled with the light variant of the ICAT reagent, oxidized thiols within the same sample are subsequently reduced and then labeled with the heavy ICAT probe. This allows peptides identical in sequence, whether they are reduced or oxidized, to be chemically identical, with the exception of a 9 Da mass shift in the oxidized peptides due to the isotopically heavy linker. Following the digestion and purification of the labeled peptides by their biotin tags, the enriched sample is run on a tandem MS/MS, where the reduced and oxidized peptides can be resolved based on their mass shift of 9 Da that is visible on the spectra. Finally, the stoichiometry of oxidized versus reduced proteins is determined by the ratio of heavy versus light peptide signal intensities (Figure 5A). oxICAT has been successfully applied for research in other model organisms, including yeast [112,113], fruit fly [114], and mammalian cells [115]. One concern associated with oxICAT is that the detection of oxidatively modified proteins that are low in abundance may be compromised by more abundant thiol-containing proteins present in the sample [116]. Additionally, ICAT reagents are not compatible for multiplexing, which limits analysis to only two samples at a time; however, other types of isobaric labeling have overcome this limitation (see following sections).

#### 2.3.2. Cysteine-Reactive Phosphate Tags

A constant challenge in thiol redox proteomics is maximizing the proteome coverage of oxidatively modified proteins, as current methods yield a coverage of hundreds to several thousand cysteine sites (approximately 1200 and 2200 sites in mouse liver and muscle, respectively [117,118]). In contrast, phosphoproteomic approaches yield a higher number of phosphorylated proteins (more than 20,000 phosphosites in HeLa and breast cancer cells [119,120]), suggesting a large divergence in the capacity of these methods to recover proteins of interest. Cysteine-reactive reagents have been modified to carry adaptable tags to improve the enrichment of cysteine-containing proteins from an extract. One such cysteine-reactive reagent is IAA, which was recently modified to carry a phosphate group that enables the labeled peptides to be purified and enriched by resins that are specific to phosphorylated proteins [121]. Through this indirect labeling method, oxidized thiol groups are essentially masked, or derivatized, with a phosphate group to improve the yield, enrichment, and specificity of cysteine-containing peptides. They can then be enriched to a much greater extent via immobilized metal affinity chromatography (IMAC) that is commonly used for phosphopeptide enrichment (Figure 5B). Xiao and colleagues adopted this approach to evaluate the cysteine redox proteome in 10 different tissues of an aging mouse model, which they termed the ‘Oximouse’ dataset [122]. From this work, they identified and determined the global oxidation stoichiometry of more than 34,000 unique cysteine sites on over 9400 proteins, demonstrating that this approach has improved throughput compared to other methods. In particular, this method identified approximately 4500 cysteine sites in mouse muscle, making it one of the more effective techniques currently for profiling thiol redox modifications in a single tissue. Additionally, this approach uses TMT labeling, which allows for multiplexing, the side-by-side comparison of samples, and more robust quantitative measurements of thiol oxidation stoichiometry (Figure 5B). While this technique initially demonstrated the ability to detect all oxidized cysteines, it could also be used to enrich for thiols that are selectively reduced to probe for a specific modification. As a recently developed technique, it has only been applied to human [121] and mouse cells [122], but has the potential to be applied to other model systems in future studies.

#### 2.3.3. RAC

An alternative approach for the enrichment of oxidatively modified proteins was introduced in 2014 by Guo and others, named resin-assisted capture (RAC) [123]. In contrast to the OxICAT and cysteine-reactive phosphate tag approaches, which process thiol-containing proteins in suspension and enrich them before MS/MS, the RAC method uses thiol-reactive resin to covalently capture reversibly oxidized thiols (nascent free thiols, Figure 5C) prior to additional processing. These nascent free thiols can be generated by different means, such as reducing all reversibly oxidized thiols with a reducing agent such as DTT, as shown in Figure 5C. As an indirect method, RAC has the versatility to profile specific modifications such as glutathionylation or nitrosylation by using specific reagents to selectively reduce them [98]. Nitrosylated proteins can be enriched by RAC using ascorbate to selectively reduce SNO groups to a free thiol. Likewise, the enzymatic reduction of SSG by glutaredoxin 1 enables RAC-mediated enrichment of glutathionylated peptides. By directly capturing thiol-containing proteins with the resin first, the sample complexity and recovery of non-specific proteins are reduced. This improves the efficiency of protein digestion and the incorporation of isobaric tags onto the remaining cysteine-containing peptides (Figure 5C). When RAC is coupled with isobaric tandem mass tag (TMT) labeling (RAC-TMT), changes in the redox state of the oxidatively modified proteins can be evaluated quantitatively. As in the approach described above (Section 2.3.2), RAC-TMT quantification takes advantage of the reporter ion intensity values to precisely measure how much oxidized peptides are present in the samples to determine the redox stoichiometry (Figure 5C). Previous work from our group has demonstrated the efficacy of RAC-TMT in different model systems, including mouse muscle [118], mouse macrophages [86,109], and cyanobacteria [124]. Recently, RAC-TMT was able to uncover more than 4000 glutathionylated sites [109], suggesting that this method’s coverage is comparable to that of cysteine-reactive phosphate tagging. By identifying at least several thousand cysteine sites with this approach, RAC is a valuable method for the field, as this coverage can provide greater detail about the redox environment.

#### 2.3.4. Thiol Reactivity Profiling

While the methods described above identify and quantify thiol oxidation, a different approach has been developed to analyze the reactivity of thiol groups. This method, named quantitative thiol reactivity profiling (QTRP) [125], is a derivation of the isotopic tandem orthogonal proteolysis-activity-based protein profiling (isoTOP-ABPP) [126,127] (Figure 5D). Fu and colleagues adapted the isoTOP-ABPP approach to create a more accessible workflow that can use commercially available reagents. Briefly, a thiol reactive probe called IPM is used to label all free thiol groups on digested peptides. IPM then reacts with either a light or heavy isotopically labeled azido-biotin reagent via a copper-catalyzed click chemistry reaction. Labeled peptides are enriched by streptavidin beads and are released through a photo-cleavable linker for detection by LC-MS/MS. By quantifying the heavy-to-light ratio, users can quantify how much free thiol is available following treatment compared to the control. While this method is presented as a 2-plex in Figure 5D, it can be adapted for use with multiplexed isobaric tags to profile multiple samples simultaneously [128]. With QTRP, users can quantitatively measure the extent of change in the reactive cysteine proteome when perturbed. QTRP has been applied to a variety of studies, including probing for the reactivity of thiols with drug-derived or natural electrophilic metabolites as well as hydrogen peroxide in human cells [128,129,130]. While QTRP is indicative of what free thiols are reactive to a given substrate, it does not resolve the state of thiols that are not detected (unreactive to labeling reagent) by this method. As a result, methods that profile for oxidatively modified cysteines may still be necessary as a complementary approach to QTRP [131]. Since some cysteine residues can be buried within a protein, they may not be accessible for labeling by IPM that occurs under native conditions and could be wrongfully interpreted as oxidized. QTRP is also not representative of the physiological state, as free thiols are likely to encounter other oxidants besides those that are similar to IPM, each of which will have different degrees of reactivity. Therefore, it is also important to use caution when considering the relevance of QTRP data as the reactivity observed under specific experimental conditions may not accurately represent physiological conditions [132]. 

### 2.4. Caveats of Current Quantitative Approaches

The methods described in this review have proven to be useful for quantitatively measuring thiol oxidation; however, they do possess some limitations that are discussed below. Following the selective reduction of oxidized thiols, it is possible that the nascent free thiols can later be re-oxidized during subsequent sample processing steps to form inter- or intra-molecular disulfides. This is especially a concern if there is a change in pH that enhances the reactivity of free thiols to form disulfides [133]. This is a potential issue for all methods using selective reduction concepts, such as the RAC, OxICAT, and cysteine-reactive phosphate tagging methods. In the case of RAC, a small concentration of DTT (<1 mM) was retained in the buffer during the capture step to prevent such issues and to ensure high recovery of formerly oxidized peptides. To protect against the unfavorable oxidation of proteins during sample processing, it is important to block all native free thiols with an alkylating agent such as N-ethylmaleimide (NEM) to prevent their oxidation. Incorporation of trichloroacetic acid (TCA) has also been used to quench thiol oxidation, as it creates an acidic environment that induces denaturing conditions and prevents thiol-disulfide exchange [134]. Another technical issue is the introduction of artefactual oxidation into the samples during long-term storage, where tissues or cell pellets can be subjected to natural oxidation over time, even when stored at −80 °C. Both issues can compromise the accurate determination of occupancies and would warrant further investigation. The use of isobaric tags for quantification, such as TMT or iTRAQ, is another source of limitation. Reporter ion intensities are only used to quantify the relative amounts of peptides across samples and conditions. However, measurements of reporter ion intensities are impacted by co-eluting peptides that are fragmented together with peptides selected as precursors in a specific isolation window in MS/MS [135,136,137]. This leads to ratio compression, where the reporter ions of coeluting peptides interfere with the signal of each channel and cause users to underestimate the quantitative differences between samples and the accuracy of the method [138,139]. Another concern with isobaric labeling strategies is their impurities, which lead to channel ‘crosstalk’, where reporter ion intensities of one channel can contribute to quantitative signals in another channel [138]. To address this issue, algorithms have been developed to deconvolve and reduce the effect of ‘crosstalk’ in a dataset.

## 3. Applications of Thiol Redox Proteomics

The continuous improvements in thiol redox proteomics have increased the field’s knowledge about the role of thiol-based redox reactions in the physiology of a cell under stressed or steady state conditions. Incorporation of stoichiometric measurements in redox proteomics datasets has improved our understanding of the extent of oxidatively modified proteins. By identifying Cys sites that have more pronounced redox stoichiometries, additional experiments can further investigate and provide insight into the physiological significance of these sites. Some current and promising future applications of quantitative redox proteomics are described below to show that quantitative thiol redox proteomics can help to advance the field of redox biology and improve our understanding of the dynamics of the thiol redox network.

### 3.1. Integrative Studies 

One emerging aspect of thiol redox proteomics is its incorporation into larger multi-omic studies to better understand molecular mechanisms and pathways. In a mouse model, investigation into the toxicological effects of cadmium exposure found that the liver is most susceptible to oxidative stress due to mitochondrial dysfunction. Using ICAT-based mass spectrometry, more than 1200 cysteine-containing peptides from more than 500 proteins were oxidized more than 50% compared to the control [140]. Pathway analysis found that amino acid metabolism was significantly perturbed, and metabolomic profiling found that more than 500 small molecules are significantly affected, including those associated with amino acid metabolism [140]. Through this bipartite approach, a variety of proteins and small molecules were identified as cadmium-sensitive factors that can disrupt the progression of metabolic pathways. In a more recent study, a combination of phosphoproteomics and thiol redox proteomics were used to uncover an elaborate signaling network in an adipocyte model following the inhibition of antioxidant defenses. Interestingly, oxidative stress inhibited the activity of the regulatory kinase Akt, despite phosphoproteomic data indicating that it was active. This multi-omics approach identified two cysteine residues, C60 and C77, that are sensitive to oxidative stress and prone to forming a disulfide that plays a role in Akt activation at the plasma membrane [141]. Implications of this study suggest that redox and phosphorylation signaling act together in a combinatorial manner to regulate Akt and potentially other pathways. Taken together, these studies demonstrate the power of integrative, multi-omic studies to uncover complex physiologically relevant pathways. In future studies, the integration of PTM stoichiometry can provide greater insight into the spectrum of PTMs that exist for a protein and how they correlate with specific pathways. 

As knowledge of redox-sensitive cysteine residues continues to increase, the development of bioinformatic tools has led to the prediction of additional sites that are potentially oxidatively modified. Using experimentally verified redox-sensitive cysteine-containing peptides from human, mouse, yeast, and *E. coli* datasets, three features of these sequences were selected. The sequential distance to nearby cysteines, position-specific scoring matrix profile, and the predicted secondary structure of flanking residues were used as criteria to develop a de novo prediction program to identify novel sites [142]. By analyzing primary sequences, this algorithm overcomes the challenge of performing analysis that is dependent on structural data, which are not widely available. Additional studies have continued to expand these predictive methods and are described elsewhere [143,144]. Interestingly, sequence-based profiling has been used to predict redox-sensitive disordered regions. This work has shown that many disordered regions contain redox-sensitive sites (more than 5% in yeast and nearly 30% in humans), and it is thought that redox modifications in these domains regulate structural conformations [145]. Together, these methods show the potential to expand the landscape of redox sensitive thiols and provide insight into the additional roles of thiol modifications in protein function.

### 3.2. Structural Insight

As mentioned above, redox-based PTMs can alter the structural conformation of a protein; therefore, it is important to consider how redox-sensitive cysteines factor in protein structure [146]. A recent study investigated how structural features of cysteine sites based on aspects such as pKa value, residue surface accessibility, and hydrophobicity are associated with the site occupancy of oxidative modifications under basal conditions. Interestingly, cysteine sites with higher occupancies were found to be localized to loop and coil structures, while low occupancy sites were typically found in α-helices or β-sheets [109]. This suggested that the oxidation of cysteine sites may be important for supporting stability in the flexible domains of proteins and the regulation of the surface properties of proteins. Indeed, another report found that cysteine residues 60 and 77 form a disulfide linkage under oxidative stress conditions, which stabilize Akt interactions with PIP3 at the plasma membrane and prolong Akt signaling [141]. Additionally, the basal state redox proteomics study found that many high-occupancy sites under basal conditions are enzymatic active sites [109], suggesting that this correlation may be informative for future studies that aim to distinguish between active and regulatory site cysteines.

Oxidative modification occupancy data may also be informative for profiling disulfides in a protein, which are important for regulating redox sensitivity as well as protein structure and function [147,148]. Comparative analyses based on oxidation occupancy in thiol redox proteomic studies and any known or inferable structural disulfide characteristics may yield insight into disulfide dynamics. Cysteines that form structural disulfides are often assumed to be retained in fully oxidized forms as disulfides and less likely in the reduced state [148], giving them the appearance of being inert; however, they may function as allosteric regulatory sites [149]. Under physiological conditions, disulfide formation is dependent on variables such as protein function, interaction partners, subcellular compartment, and pH. This means that for disulfides that are identified structurally, the frequency (occupancy) of the disulfide at the physiological level may only be partially, and not totally, oxidized. A study of total thiol oxidation under basal conditions identified a variety of proteins with disulfide-associated cysteine sites that had variable oxidation occupancies. Protein disulfide isomerases A3 and A4, as well as thioredoxin 12, were among the proteins identified as having total oxidation occupancies of 73.9%, 10.6%, and 13.3%, respectively [109]. This was a surprising finding given that these are localized to the endoplasmic reticulum, a compartment that is a highly oxidative environment. More studies are needed to evaluate whether these are structural or regulatory disulfides; however, these findings point to the distribution of thiol oxidation occupancies as being more dynamic, rather than binary (i.e., totally reduced or oxidized). While the oxidation occupancies of disulfides may not necessarily be in agreement with the structural information, they help researchers to better understand the significance of these sites. Beyond disulfides, other forms of thiol modification can be investigated with visual tools at the molecular level to understand their impact.

Simulation techniques have the potential to provide insight into the relationships between cysteine oxidation and its effect on protein function. By using quantitative redox proteomics to identify highly oxidized cysteines, these sites can be the subject of more intensive in silico studies that model the effects of thiol oxidation on protein structure [150]. Previous work has found that apolipoprotein E isoform 3 (ApoE3) directly interacted with nitric oxide synthase 1, which leads to nitrosylation at Cys112 of ApoE3 [151]. Using the crystal structure of ApoE3, modeling of C112SNO showed that the ApoE3 structure was altered such that the receptor binding domain changed its conformation. This abrogated ApoE3’s interaction with the low-density lipoprotein (LDL) receptor and affected the regulation of lipid metabolism. Another example is the collapsin response mediator protein 2 (CRMP2), which is critical for many aspects of embryonic development, including axon growth in the brain [152]. CRMP2 is regulated by PTMs such as phosphorylation and oxidation, which control its biological activity and interactions with other proteins [153]. Using mass spectrometry, an intermolecular cysteine, 504-cysteine 504 disulfide, that forms between two CRMP2 monomers was identified as an oxidative modification [154]. Molecular dynamics simulations demonstrated that this disulfide in the flexible C-terminal region of CRMP2 leads to structural changes that impact phosphorylation status under oxidizing or reducing conditions [155]. As a result, these findings contribute to the concept of a thiol-disulfide switch, where phosphorylation is dependent on the redox status of CRMP2. Simulation integration with redox proteomics was also shown with Akt, where in silico modeling was used to study how the formation of an intramolecular disulfide between cysteine residues 60 and 77 impacts Akt function. Molecular dynamics simulations demonstrated that the cysteine-60/77 disulfide in wild-type Akt stabilizes loop regions 1 to 3, which are necessary for interaction with PIP3 via nine contacts observed in docking simulations [141]. A cysteine-60/77-Serine mutant was also modeled to mimic the loss of the disulfide, where only three contacts were transiently formed, suggesting that the disulfide bond formation is important for Akt recruitment to PIP3 [141]. These reports show that in silico modeling provides mechanistic insight into how different oxidative modifications impact protein function. In future studies, redox proteomics and stoichiometry will help to continue identifying critical redox-sensitive cysteine sites to determine their role in protein structure. 

### 3.3. Potential Health and Clinical Applications 

Thiol redox proteomics has improved our understanding about redox-sensitive proteins and how the redox state is perturbed by a mutation or in response to stimuli. Consequently, multiple aspects of thiol redox proteomics have garnered interest in the clinical field, such as diagnostics, biomarker identification, and drug development [156]. Since oxidative stress typically accompanies many types of diseases, identifying key proteins that have elevated levels of oxidation in sick versus healthy patients may serve as biomarkers for specific disease diagnostics. A recent study identified human serum albumin as a putative biomarker for diagnosing and monitoring type 2 diabetes progression. Specifically, Paramasivan et al. used discovery proteomics to profile for the trioxidation (sulfonylation) of human serum albumin in healthy and type 2 diabetes patients and found that more trioxidized forms of the albumin were present in the diabetic patients [157]. As a follow-up, targeted proteomics narrowed down 3 out of 13 trioxidized human serum albumin cysteine sites (Cys 34, 265, and 487) that were reliably detected and quantified in diabetic and healthy patients [157]. This analysis confirmed the trend observed in the discovery proteomics approach and supports trioxidation of human serum albumin as a strong candidate biomarker for type 2 Diabetes. Thiol redox proteomics has also been powerful for predicting and monitoring the progression of Chagas disease caused by *Trypanosoma cruzi* infection, which leads to heart disease or failure. To control the pathogen, immune and non-immune cells increase the production of reactive oxygen and nitric oxide. However, failure to resolve the infection can cause prolonged exposure to these oxidants, which leads to oxidative stress and the modification of host proteins. Using peripheral blood mononuclear cells derived from patients that are seronegative healthy and seropositive asymptomatic or symptomatic for Chagas disease, SNO modifications were analyzed to identify proteins with significantly altered nitrosylation profiles as indicators of disease state [158]. From this study, nitrosylation of proteins such as KRT1, PNP, and ACTB were identified as biomarkers that distinguish sick from healthy patients and could be used in a panel with high confidence to diagnose and monitor disease progression [158]. Beyond diagnostics and biomarker discovery, the development of drugs that target redox sensitive sites is another avenue of thiol redox proteomics research that may impact treatments used in clinics.

Since thiol redox reactions are an underlying part of the mechanisms that lead to disease, using drugs that function as antioxidants may prove to be an effective therapeutic intervention to counter oxidative stress. The mitochondrial-targeted synthetic peptide SS-31, also known as elamipretide, is one such drug that has been shown to reduce H_2_O_2_ production [159] and improve ATP production [160]. The benefits of SS-31 have been investigated in a variety of studies, including those that investigate oxidative stress as a cause of aging and exercise intolerance. In an aging mouse model, where skeletal muscle function and mitochondrial capacity is reduced, the administration of SS-31 reversed age-related mitochondrial dysfunction and improved ATP production as well as muscle function [161]. An investigation into the global profile of the glutathionylation of cysteine in young and aged mice treated with or without SS-31 was also performed. Interestingly, the SS-31 treatment of aged mice resulted in a glutathionylation profile that was very similar to young mice, and the proteins that experienced the most significant change were associated with the TCA cycle [161]. The effects of SS-31 on aging mouse heart muscle have also been studied, where SS-31 treatment reduced ROS production, improved the function of cardiomyocytes, and also significantly improved exercise performance compared to untreated mice [162]. The average cysteine glutathionylation occupancy was measured as a metric of the redox state, where old mice treated with SS-31 had a more reduced redox environment (5.9%) compared to untreated aged mice (7.1%) and was comparable to young mice (5.3%) [162]. These studies demonstrate that mitochondria, which are major sources of oxidative stress, can be targeted by therapeutics to help normalize the redox state and restore the function of aging or stressed organs. 

## 4. Conclusions and Outlook

Thiol redox proteomics has dramatically improved our understanding of redox-dependent biological and molecular processes, as well as deepened our appreciation for these mechanisms in a variety of contexts. From a single thiol group on the amino acid cysteine comes a diverse array of redox-based PTMs, each of which has distinct chemical properties (Figure 1) that give rise to cysteine’s utility in many aspects, such as the regulation of catalytic activity, protein-protein interactions, or the regulation of protein conformation (Figure 2). To better understand how these and other aspects are controlled, it is important to have sensitive methods that can accurately identify and quantify redox PTMs (Figure 5) on cysteines that are either labile or have low abundance. The methods described in this review provide a way for researchers to grasp the extent of thiol redox modifications that are present in a model system. Furthermore, occupancy calculations (Figure 4) are informative for determining the stoichiometry for identifying critical redox-sensitive sites. While only the total oxidation of thiol groups was considered during the discussion of quantitation in this review, studying each individual type of thiol redox PTM is equally important. In future studies, it will be useful to study a panel of thiol redox PTMs simultaneously to understand what modifications make up observed total thiol oxidation. 

As the identification of more thiol redox-sensitive proteins continues to increase, their importance in many biological processes is beginning to be appreciated. We discussed some examples where thiol redox reactions played critical roles as allosteric regulators or switches of protein function, which may become a more pronounced effect of thiol redox PTMs in the future. When analyzing the thiol redox network following a perturbation, the significant effects of thiol redox signaling can occur on a microscopic level and could go unnoticed if focus is directed towards the macroscopic level (Figure 3). In the absence of a major shift in the redox state (as indicated by a transition from a more oxidizing to a more reducing environment or vice versa), it is possible for specific circuits within thiol redox networks to be perturbed. This can be inferred by analyzing the occupancy of specific constituents of a pathway that show significant differences between control and experimental conditions. Quantitative thiol redox proteomics holds great potential to guide researchers to investigate specific redox-dependent mechanisms associated with a disease and aid in the design of different therapeutics to help treat those diseases.
